# LMO2 is required for TAL1 DNA binding activity and initiation of definitive haematopoiesis at the haemangioblast stage

**DOI:** 10.1093/nar/gkx573

**Published:** 2017-06-30

**Authors:** Vesna S. Stanulović, Pierre Cauchy, Salam A. Assi, Maarten Hoogenkamp

**Affiliations:** Institute of Cancer and Genomic Sciences, College of Medical and Dental Sciences, University of Birmingham, Birmingham B15 2TT, UK

## Abstract

LMO2 is a bridging factor within a DNA binding complex and is required for definitive haematopoiesis to occur. The developmental stage of the block in haematopoietic specification is not known. We show that *Lmo2^−/−^* mouse embryonic stem cells differentiated to Flk-1^+^ haemangioblasts, but less efficiently to haemogenic endothelium, which only produced primitive haematopoietic progenitors. Genome-wide approaches indicated that LMO2 is required at the haemangioblast stage to position the TAL1/LMO2/LDB1 complex to regulatory elements that are important for the establishment of the haematopoietic developmental program. In the absence of LMO2, the target site recognition of TAL1 is impaired. The lack of LMO2 resulted in altered gene expression levels already at the haemangioblast stage, with transcription factor genes accounting for ∼15% of affected genes. Comparison of *Lmo2^−/−^* with *Tal1^−/−^* Flk-1^+^ cells further showed that TAL1 was required to initiate or sustain *Lmo2* expression.

## INTRODUCTION

LIM only 2 (LMO2) was originally identified through its homology to LMO1 and was shown to cause T-cell acute lymphoblastic leukaemia, as a consequence of chromosomal translocations involving *LMO2* and the T-cell receptor genes (1). The protein consists of two LIM domains, which mediate protein-protein interactions. The first LMO2 protein complex was characterised in the erythroid lineage and besides LMO2 contains the transcription factors TAL1, E2A, LDB1 and GATA1. LMO2 links the DNA binding TAL1-E2A dimer with GATA1, as well as with LDB1 (2). LDB1 self-associates to form trimeric structures (3), thereby nucleating multiple LMO2 complexes and facilitating long range chromatin interactions (4,5). Further research in erythroid cells identified more components of this complex and their dynamic changes during differentiation (6). In addition, other variants of the DNA binding factors within the LMO2-containing protein complexes have been reported, where GATA1 is replaced by GATA-2 (7,8), GATA3 (9) or a second TAL1-E2A dimer (10), as well as interactions with a number of other transcription factors (7,11).

Blood development in the mammalian embryo occurs in three temporally overlapping waves. In the mouse embryo, the first blood cells appear in the extraembryonic yolk sac around embryonic day (ED) 7.5, consisting of primary erythroblasts, macrophages and megakaryocytes (12,13). A day later a second wave starts, also at the yolk sac blood islands, which produces definitive erythro-myeloid progenitors and progenitors with lymphoid potential (14,15). The yolk sac derived progenitors transiently populate the foetal liver and circulation, prior to the establishment of the ‘adult’ haematopoiesis (14). The final wave starts at ED10.5 and is characterised by the emergence of the haematopoietic stem cells (HSCs), which are at the base of the multilineage haematopoietic hierarchy found in adults. Whereas the first two waves originate from the yolk sac, the HSCs originate from the aorta-gonad-mesonephros region of the developing embryo (16,17). These stem and progenitor cells migrate to the developing liver, where expansion occurs prior to relocating to their final place in the bone marrow (18).

During development, emerging blood progenitors are the product of a cellular differentiation process, which starts by the specification of haemangioblasts (HBs). HBs are mesoderm-derived progenitors that have the potential to give rise to vascular smooth muscle cells, endothelial cells of the early vasculature and, via a transient stage of haemogenic endothelium (HE), to haematopoietic stem and progenitor cells (19–22). HE undergoes a process termed endothelial-to-haematopoietic transition, generating primitive or definitive haematopoietic progenitors (23,24). LMO2 is crucial for both primitive and definitive haematopoiesis (25). Homozygous deletion of *Lmo2* leads to embryonic lethality around ED10.5, due to a complete lack of yolk sac erythropoiesis (26). However, macrophages were observed at this stage, indicating that haematopoietic progenitors were produced, albeit with a block in erythroid differentiation. Through the analysis of chimeric mice, it was shown that *Lmo2*^−/−^ cells did not contribute to definitive haematopoiesis (25). In addition, these cells did not contribute to the endothelial component of larger blood vessels, such as the dorsal aorta (27). Chimeras with a high proportion of *Lmo2*^−/−^ cells displayed disrupted vascular organisation. The absence of Lmo2^−/−^ cells from the dorsal aorta at the time of the emergence of the HSCs suggests that they do not participate in the formation of HE at this site. However, to date the precise nature of this block and the target genes depending on LMO2 are not known.

The function of the LMO2 binding partners LDB1, TAL1, GATA1/2 has been studied in knock out mouse models. *Ldb1^−/−^* embryos die between ED8.5 and ED9.5, *Tal1^−/−^* embryos at day 9.5, *Gata2^−/−^* embryos die at ED10.5, whereas *Gata1^−/−^* embryos die at ED11.5. The time of lethality is paralleled with the time of the block in differentiation. *Ldb1^−/−^* mice have blocked vasculogenesis and the number of Flk-1^+^ HBs is reduced to half of the level found in WT (28,29). *Tal1^−/−^* embryos have developed vasculature but lack primitive blood islands in the yolk sack (30). *Gata1^−/−^* have developed blood islands that give rise to primitive macrophages and erythroblasts that are unable to further mature to erythrocytes (31). *Gata2^−/−^* embryos only have a mild reduction in the number of primitive haematopoietic progenitors and primitive erythrocytes, but fail to develop definitive haematopoiesis (32). Experiments with chimeric mice showed that TAL1, LMO2 and GATA2 are instrumental for definitive haematopoiesis and the formation of all the blood lineages (27,32,33). This is in line with the finding that *in vitro* differentiated *Tal1^−/−^* ES cells give rise to Flk-1^+^ HBs (20), but fail to generate HE and that GATA2 is required for the specification of the HE in the dorsal aorta (34).

In this study, we investigate the role of LMO2 during early haematopoietic development, using an established *in vitro* differentiation system. In this system, mouse embryonic stem (ES) cells are differentiated towards the haematopoietic fate through defined developmental stages (17,20). We show that *Lmo2^−/−^* ES cells can give rise to Flk-1^+^ HBs, but have a reduced ability to generate the HE, which further fails to give rise to definitive haematopoietic progenitors, but inefficiently produces primitive haematopoietic progenitors. Combining genome wide expression, ChIP and chromatin accessibility assays, we show that in Flk-1^+^ cells the LMO2 complex regulates many genes encoding transcription factors and genes important for embryonic development and differentiation. Changes in gene expression are already observed at this stage, although upregulation of target genes becomes particularly clear at the following HE1 stage. Using Tal1^−/−^ cells we discriminate between the effects of loss of TAL1 versus LMO2 and identify TAL1 as the initiator of the gene expression program. However, in *Lmo2^−/−^* cells many TAL1 peaks are found at novel positions, with TAL1 binding only retained at the highest affinity sites found in WT, but at a lower level. This indicates that LMO2 complex formation is required for correct target site recognition by TAL1 and for execution of the haematopoietic program.

## MATERIALS AND METHODS

### Cell culture

The CCB, *Lmo2^−/−^* and *Tal1^−/−^* ES cell lines and their culture conditions have been described before (26,35,36). ES cells were maintained on a layer of primary mouse embryonic fibroblasts (MEFs) in KnockOut DMEM (Life Technologies), supplemented with 15% EScult FBS (Stemcell technologies), 100 U/ml penicillin, 100 μg/ml streptomycin, 25 mM Hepes buffer, 1× Glutamax (Life Technologies), 1× non-essential amino acids (Sigma), 0.15 mM monothioglycerol (MTG), 10^3^ U/ml ESGRO (Millipore). Cells were passaged every other day and tested for mycoplasma contamination. Prior to the start of differentiation ES cells were passaged in the absence of MEFs on gelatin-coated dishes and after 24 h the medium was replaced with IMDM containing the same additives as for ES cell maintenance. After 48 h differentiation cultures were started by diluting single cell suspensions to 3.5 × 10^4^ cells/ml in *in vitro* differentiation (IVD) medium, consisting of IMDM, supplemented with 15% heat-inactivated FBS (Gibco 10500), 100 U/ml penicillin, 100 μg/ml streptomycin, 0.15 mM MTG, 50 μg/ml ascorbic acid, 180 μg/ml human transferrin (Roche). Cell fractionation has been described before (37,38). Briefly, embryoid bodies were dissociated with trypsin and Flk-1^+^ cells were isolated by magnetic cell sorting (MACS) using biotinylated Flk-1 antibody (eBioscience 13-5821), antibiotin microbeads (Miltenyi Biotec 130-090-485) and MACS LS columns (Miltenyi Biotec 130-042-401) according to manufacturer's instructions. For further differentiation of Flk-1^+^ cells into haemogenic endothelium and haematopoietic progenitors, cells were cultured in gelatin-coated culture flasks in IMDM, supplemented with 10% heat inactivated FBS (Gibco 10500), 20% D4T conditioned medium (24), 100 units/ml penicillin, 100 μg/ml streptomycin, 0.45 mM MTG, 25 μg/ml ascorbic acid, 180 μg/ml human transferrin (Roche), 5 ng/ml VEGF (PeproTech 450-32), 10 ng/ml IL-6 (PeproTech 216-16). Cell populations were analysed using Kit-APC, Tie2-PE, CD41-PECy7 and CD45-APC antibodies (eBioscience 17-1171, 12-5987, 25-0411, 17-0451) by FACS (BD FACSAria Fusion) and flow cytometry (Beckman Coulter CyAn ADP).

### Expression analysis

RNA was isolated using an RNeasy Mini Kit (QIAGEN) according to the manufacturer's protocol, after which concentration and quality were determined on a NanoDrop 2000 UV-Vis Spectrophotometer (Thermo Scientific). For cDNA synthesis, typically 2 μg RNA was reverse transcribed using Oligo(dT)_12–18_ primer and SuperScript II Reverse Transcriptase (Life Technologies), after which gene expression was measured by quantitative PCR (qPCR), using SYBR green (Thermo Fisher 4309155) on an ABI 7500 Real-Time PCR System with primers listed in Supplementary Table S1. Quantitation was carried out using a standard curve generated by a five-point 4-fold serial dilution of cDNA. RNAseq libraries were prepared using the TruSeq Stranded mRNA Sample Preparation Kit (Illumina), using 1 μg total RNA per sample. Three independent libraries were prepared of Flk-1^+^ and two of HE1. Libraries were run on an Illumina HiSeq 2500 sequencer. At least 30 million 100nt paired-end reads were acquired per library.

### Western blotting

Crude nuclear extracts were prepared as described before (39) and separated on 4–12% gels (Novex, Life Technologies). Proteins were transferred to nitrocellulose membranes, blocked and incubated overnight with primary antibody, followed by 1 h incubation with the appropriate secondary antibody. Primary antibodies raised against TAL1 (Santa Cruz sc-12984X), Ldb1 (Abcam ab96799), LMO2 (R&D systems AF2726), β-actin (Sigma A1978) were used at a final concentration of 1 μg/ml and secondary antibodies were IRDye 680RD or 800RD (Li-Cor), which were used at a 1:2000 dilution. Fluorescence was detected with an Odyssey CLx Imager (Li-Cor). Quantitation of bands was performed by densitometry, using the Odyssey v3.0 software.

### DHS mapping

Genome wide DNaseI hypersensitivity (DHS) mapping was performed as described before (39,40). DNA from *in vivo* DNase1-treated cells was isolated and approximately 10 μg was size fractionated on an agarose gel. DNA fragments in the range of 100–600 bp were isolated using QIAGEN gel purification columns and library preparation was performed according to the Illumina library preparation protocol. Sequencing was performed on an Illumina Genome Analyser.

### Chromatin immunoprecipitation

Chromatin Immunoprecipitation (ChIP) assays were performed as described (38). Isolated Flk-1^+^ cells were crosslinked with 1% formaldehyde for twelve minutes at room temperature. Nuclei were isolated and the chromatin was sonicated for 10 cycles of 30 s on 30 s off at 4°C, using a Q800R sonicator (Active Motif). Immunoprecipitation was carried out using protein G magnetic beads (Dynal) with the antibodies as described for western blotting (1.5 μg antibody/10μl of beads). After elution, crosslinks were reversed overnight at 65°C, after which the DNA was isolated using Ampure PCR purification beads and analysed by qPCR (for primers see Supplementary Table S1) or libraries were prepared using the Illumina protocol with indexed primers. Libraries were run on an Illumina HiSeq 2500 in rapid run mode.

### Genome wide data analysis

Global analysis of the genome wide data was performed on usegalaxy.org (41–43) and on the University of Birmingham High Performance Computing cluster.

Acquired RNAseq reads were mapped to the mouse genome (GRCm38/mm10) using TopHat 2.0.9 (44). Transcripts were assembled using Cufflinks 2.0.0 based on the reference genome with quartile normalisation and effective length correction. A combined gtf file was produced using Cuffmerge 2.1.1 and used for determination of the differential gene expression using Cuffdiff 2.0.1. Principle component analysis and hierarchical clustering were performed on all RNAseq samples to assess inter-sample variation (Supplementary Figure S3C and S3D). Gene differential expression and FPKM gene expression files were used to select the gene IDs that were significantly differentially expressed, with *P* < 0.05, longer than 200 bp and FPKM >10 in at least one of the samples. Heat maps, Hierarchical Clustering were computed by MultiExperiment Viewer v4.9.0 based on Pearson correlation with complete linkage. The number of clusters was determined by Self Organizing Tree Analyses. Gene ontology enrichment was performed using DAVID 6.7 (45) and GREAT (46). GO terms for biological processes with *P*-value <0.05 were considered significant, categories with redundant terms were filtered out, and the first 10 were presented.

For DHS and ChIPseq analysis, reads were mapped to the mouse genome mm10 (GRCm38) using Bowtie 1.1.2. Duplicates were removed using Picard MarkDuplicates 1.56.0 and peaks were called using MACS 1.0.1 (47) with tag size 28 and band width 200. For LMO2 WT and KO DHS unions, LMO2 WT and KO DHS summits were concatenated (48). DHS tag counts (±1000 bp) around the summits were retrieved using Homer (49). Tag counts (±100 bp) of each summit were added up, log_2_ transformed and sorted by increasing log_2_ LMO2 KO/WT fold change. For the LMO2 ChIP and corresponding heatmaps, WT LMO2 ChIP summits were sorted by descending tag counts and tag counts (±1000 bp) around summits were recovered. Heatmaps were generated with Java TreeView (50).

Genome wide data reported in this study are available from the NCBI Gene Expression Omnibus portal (GEO: GSE99938).

## RESULTS

### *Lmo2^−/−^* cells are progressively compromised in their haematopoietic potential

**Figure 1.**
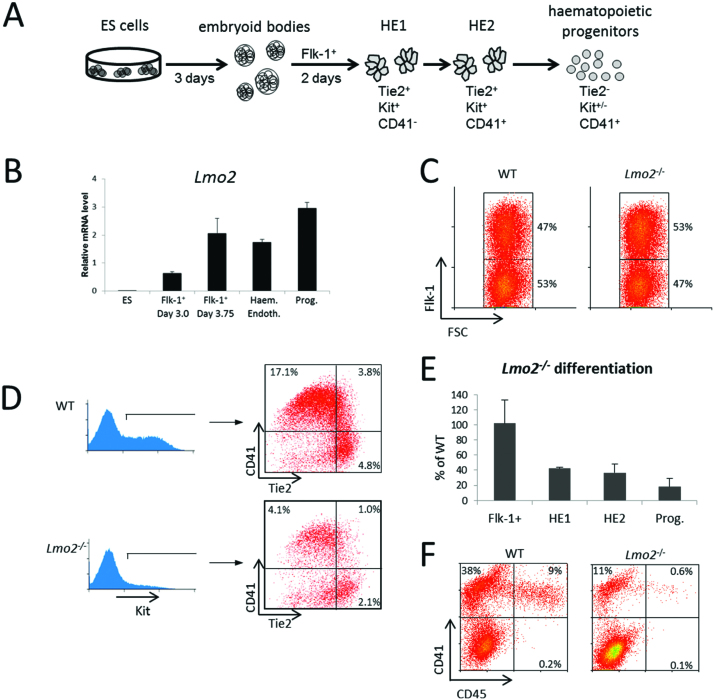
LMO2 is necessary for haematopoietic development. (**A**) Schematic representation of the ES cell in vitro differentiation system. ES cells were differentiated and Flk-1^+^ cells were isolated from embryoid bodies. These were then further differentiated in a blast culture, giving rise to haemogenic endothelial cells (HE1 followed by HE2), which subsequently generate haematopoietic progenitor cells. (**B**) Expression levels of *Lmo2* mRNA during *in vitro* ES cell differentiation, measured by qPCR. Data are the mean of at least three independent samples measured in duplicate ± SEM and were normalised to *Hprt* expression. (**C**) WT and *Lmo2^−/−^* ES cells were differentiated to day 3.75. Flk-1 surface expression was measured by flow cytometry, with prior gating for live cells using the FSC and SSC plot. (**D**) WT and *Lmo2^−/−^* cells were harvested at day 2 of the blast culture and stained for Kit, Tie2, and CD41 surface expression. Indicated percentages relate to the total number of gated live cells, using the FSC and SSC plot. (**E**) Abundance of Flk-1^+^, HE1, HE2, and progenitors in *Lmo2^−/−^* differentiation cultures, relative to WT. Data are the mean of at least three independent samples ± SEM. (**F**) WT and *Lmo2^−/−^* progenitor cells were enriched at day 3 of blast culture by resuspending non- and loosely-adherent cells and stained for CD41 and CD45 surface expression. Representative scatter plots are shown.

*Lmo2^−/−^* cells do not contribute to the definitive haematopoietic system (25,27). In order to determine at which stages the developmental block occurs, we employed an established *in vitro* ES cell differentiation system (Figure 1A) (20), using WT and *Lmo2^−/−^* ES cell lines (26). ES cells start differentiation by generating embryoid bodies (EBs). From these, haemangioblasts were enriched by isolating Flk-1^+^ (VEGFR2) cells, which were subsequently either used for analysis, or further cultured in blast medium for the formation of haemogenic endothelium 1 (HE1; Tie2^+^, Kit^+^, CD41^−^), and via haemogenic endothelium 2 (HE2; Tie2^+^, Kit^+^, CD41^+^) to haematopoietic progenitors (Tie2^−^, Kit^+/−^, CD41^+^). Expression of *Lmo2* was first detected in the early Flk-1^+^ cells, isolated at day 3.0 and was increased in Flk-1^+^ cells isolated at day 3.75. This level was maintained in HE1 and haematopoietic progenitor cells (Figure 1B). Differentiation of WT and *Lmo2^−/−^* ES cells into day 3.75 EBs showed that *Lmo2^−/−^* cells had equal capacity to generate Flk-1^+^ cells (Figure 1C and E). Further differentiation of haemangioblasts in blast medium, resulted in the generation of Tie2^+^, Kit^+^ HE. However, cultures of *Lmo2^−/−^* cells typically contained only 40% of the HE cells observed in the WT and the number of haematopoietic progenitors was further reduced to approximately 20% of WT (Figure 1D and E). Staining of haematopoietic progenitors for CD41 and CD45 at day 3 of the blast culture showed an almost complete absence of CD45^+^*Lmo2^−/−^* progenitors, indicating that these progenitors are a product of primitive haematopoiesis (Figure 1F). The expression profile of key transcription factors genes showed the WT CD41^+^, CD45^−^ progenitors expressed higher levels of *Tal1, Gata1, Gata2, Runx1, Gfi1, Gfi1b*, and *Nfe2*, whereas CD41^+^, CD45^+^ cells exhibited higher levels of *Spi1* and *Cebpb* (Supplementary Figure S1).

### RNAseq analysis reveals affected transcriptional regulators

**Figure 2.**
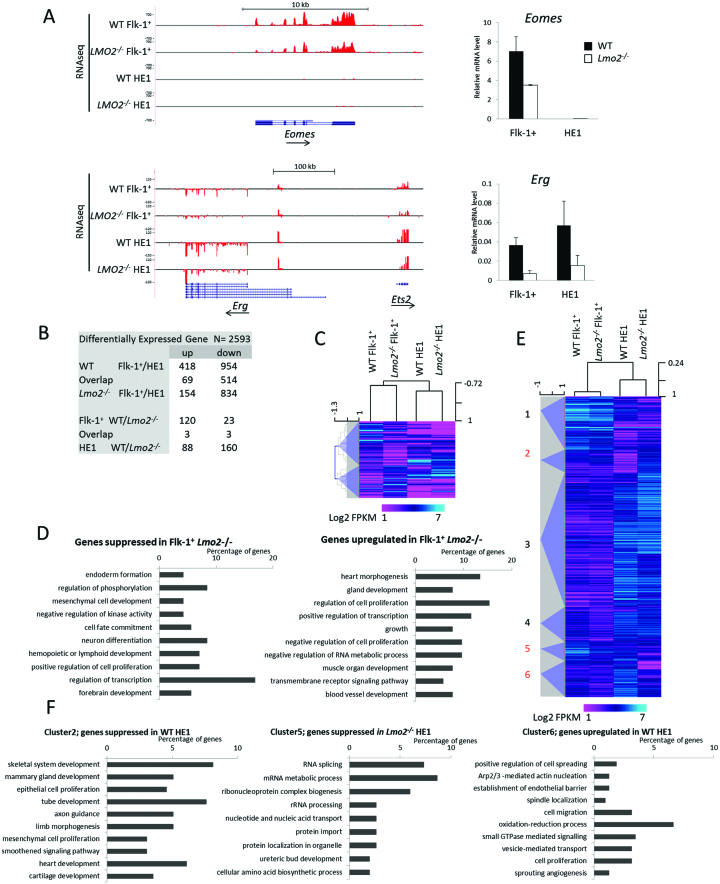
RNAseq analyses of WT and *Lmo2^−/−^* Flk-1^+^ and HE1 cells. (**A**) Screenshots from the UCSC browser illustrating the distribution of reads over the *Eomes*; *Ets2*, and *Erg* loci. Uniform y-axis scales were used for each genomic region and positive and negative values correspond to the direction of the transcripts. The screenshots are flanked by gene expression levels of *Eomes* and *Erg*, measured by qPCR. Data represent the mean of three independent samples measured in duplicate ± SEM relative to *Hprt*. (**B**) Pairwise comparison of the significantly differentially expressed genes. Shown are the total number of up or down regulated genes for each comparison and their overlap. Quantitation and analyses of RNAseq data are based on biological triplicate samples for Flk-1^+^ cells and duplicates for HE1 cells. (**C**) Heat map showing hierarchical clustering of differentially expressed genes at the Flk-1^+^ stage. (**D**) Gene ontology enrichment analyses for biological process performed on the two clusters identified in C. (**E**) Heat map showing hierarchical clustering of all differentially expressed genes. Scale bar represents colour index for the log_2_ FPKM values. Self-organizing tree analysis identified six clusters, which are numbered 1 to 6. (**F**) Gene ontology enrichment analyses for biological process was performed on clusters 2, 5, 6 identified in E. Terms were ordered according to their Modified Fisher Extract *P*-value and only terms with *P* < 0.05 were considered significant.

To investigate how the lack of LMO2 impacts on gene expression, we analysed purified WT and *Lmo2^−/−^* Flk-1^+^ cells and HE1 by RNAseq (Figure 2A, Supplementary Figures S2 and S3). Pairwise comparisons identified 2593 differentially expressed genes (Figure 2B). Differentiation from HB to HE1 was characterised by a progressive deviation of gene expression between wild-type and *Lmo2^−/−^* cells accounting for 69% (1777 genes) of the differential gene expression. At the haemangioblast stage only 143 genes were differentially expressed between WT and *Lmo2^−/−^*. Hierarchical clustering of these genes revealed that the expression of these genes is specific to the HB stage since the majority of the genes are not expressed in the HE1 cells (Figure 2C). Subsequent gene ontology analysis showed that *Lmo2^−/−^* cells failed to upregulate genes important for endoderm formation, cell fate commitment and haematopoietic development amongst others, whereas genes involved in heart and muscle development and endothelial fate were expressed at higher levels by *Lmo2^−/−^* cells (Figure 2D). Transcriptional regulators were well represented in both clusters. Within the list of transcriptional regulators down-regulated in *Lmo2^−/−^* Flk-1^+^ cells we found several transcription factors with known functions at the HB / HE1 stages. These included the endomesodermal T-box factors *T* and *Eomes*, and transcription factors important for haematopoietic development, such as *Tal1, Erg, Gata2* and *Fli-1* (Supplementary Figure S3A). Self-organizing tree clustering of all the differentially expressed genes produced six clusters (Figure 2E). Two clusters typified by higher expression in the WT cells are cluster 5 (high WT Flk-1^+^ and HE1) and cluster 6 (high WT HE1), whereas cluster 2 showed elevated expression in *Lmo2^−/−^* HE1. These three clusters were subjected to gene ontology analysis (Figure 2F). Cluster 5 is heavily dominated by terms involving translation, RNA processing and RNA metabolism, whereas cluster 6 showed cellular processes and angiogenesis. Cluster 2 on the other hand indicated alternative cell fates, such as epithelium, endothelium, muscle development and limb formation.

### Positive feedback between LMO2 and TAL1 regulates their expression levels

**Figure 3.**
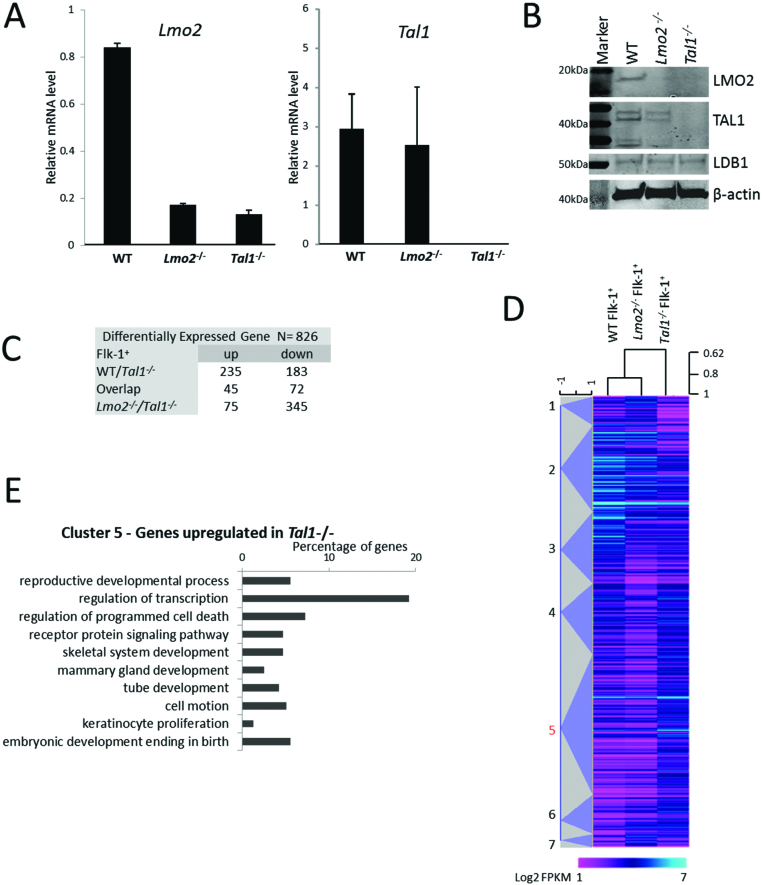
Gene expression analysis of *Tal1^−/−^* Flk-1^+^ cells. (**A**) *Lmo2, Tal1* gene expression relative to *Hprt* in Flk-1^+^ cells derived from WT, *Lmo2^−/−^* or *Tal1^−/−^* ES cells. Data represent the mean of three independent samples measured in duplicate ± standard deviation. (**B**) LMO2, TAL1, LDB1 and β-actin protein levels detected by western blotting using nuclear extract from WT, *Lmo2^−/−^* and *Tal1^−/−^* Flk-1^+^ cells. (**C**) Pairwise comparison of significantly differentially expressed genes. Quantitation and analyses of RNAseq data are based on biological triplicate samples. (**D**) Heat map showing hierarchical clustering of differentially expressed genes at the Flk-1^+^ stage. (**E**) Gene ontology enrichment analysis for biological process was performed on cluster 5 as identified in D. Terms were ordered according to their Modified Fisher Extract *P*-value and only terms with *P* < 0.05 were considered significant.

Our RNAseq data indicated that *Tal1* mRNA levels were reduced in *Lmo2^−/−^* Flk-1^+^ cells, but not in HE1 (FPKM 32.5 versus 13.5 in WT and *Lmo2^−/−^* HB respectively). Although qPCR did not detect a significant change in *Tal1* mRNA expression, we observed a reduction in TAL1 protein of >75% in *Lmo2^−/−^* Flk-1^+^ cells (Figure 3A and B), whereas the abundance of other components of the complex were unaffected (Figure 3B, Supplementary Figure S4). This finding could infer that most of the *Lmo2^−/−^* phenotype was due to the reduction in TAL1 protein. To address this we differentiated *Tal1^−/−^* ES cells. Interestingly, RNAseq analysis of *Lmo2* expression in *Tal^−/−^* Flk-1^+^ cells showed that mRNA was at background levels and LMO2 protein was not detectable, highlighting the importance of TAL1 for *Lmo2* gene activation (Figure 3A and B). The data therefore showed that *Tal1^−/−^* HBs were not only deficient for TAL1, but also for LMO2, whereas the *Lmo2^−/−^* cells retained low levels of TAL1 protein. RNAseq on the *Tal1^−/−^* Flk-1^+^ cells and the comparison with the WT and *Lmo2^−/−^* cells at this stage identified 826 differentially expressed genes (Figure 3C). Hierarchical clustering of these genes indicated that *Tal1^−/−^* HBs are more divergent from the WT than *Lmo2^−/−^* HBs (Figure 3D). Gene ontology analysis on cluster 5, which contains genes that are expressed at the highest level in *Tal1^−/−^* cells (Figure 3E), showed a large number of transcriptional regulators. In addition, it showed upregulation of early developmental processes and alternative cell fates, such as epithelium, skeletal, keratinocyte and gland development. This indicates that the *Tal1^−/−^* HBs displayed a developmentally earlier defect than *Lmo2^−/−^* HBs, as they failed to suppress the somatic mesoderm and neural crest gene expression program.

### *Lmo2* deficiency leads to the selective loss of accessible chromatin sites

**Figure 4.**
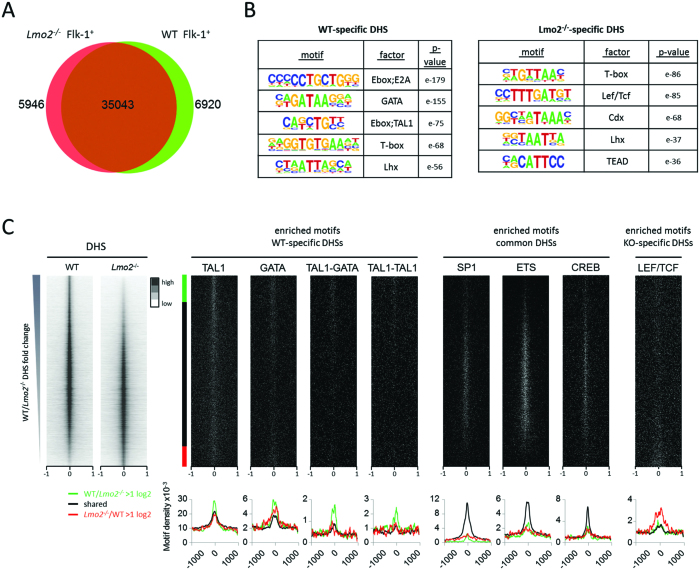
Analysis of DNaseI chromatin accessibility. (**A**) Venn diagram comparing DNaseI hypersensitive sites of WT and *Lmo2^−/−^* Flk-1^+^ cells. (**B**) *De novo* motif analysis showing enriched motifs within the WT-specific and *Lmo2^−/−^*-specific DHSs, their DNA-binding factors and associated *P*-values. (**C**) Heat maps showing DHSs ranked according to their WT/*Lmo2^−/−^* ratio showing a window from –1 kb to +1 kb around the centre of the DHSs. The left two panels show the DHSs from the WT and *Lmo2^−/−^* Flk-1^+^ cells. Beside it a guide indicates which positions show a WT/*Lmo2^−/−^* ratio of >log_2_ (green; 6921 sites), *Lmo2^−/−^*/WT ratio of >log_2_ (red; 5050), or a ratio of <log_2_ (black; 35 964). The panels to the right show the presence of the indicated motifs, using the same ranking and window from –1 kb to +1 kb around the centre of the DHSs. Below each binding motif matrix an average profile represents the distribution of the WT-enriched (green), common (black) and *Lmo2^−/−^*-enriched (red) motifs.

In order to understand how the accessibility of *cis*-regulatory elements, such as promoters and enhancers, was influenced by the absence of LMO2 we performed DNaseI hypersensitive site (DHS) mapping on WT and *Lmo2^−/−^* Flk-1^+^ cells. Cells were purified and subjected to *in vivo* DNaseI digestion, after which DNA fragments were isolated and processed for sequencing. We identified 40 989 DHSs in the WT cells and 41 963 DHSs in *Lmo2^−/−^* cells. The intersection of these two datasets showed that the majority of the DHSs overlap (Figure 4A). However, the analysis also showed that a large number of DHS present in WT cells was lost in *Lmo2^−/−^* cells. *De novo* motif analysis of this population of DHSs (Figure 4B) showed a strong enrichment for class I bHLH E-box (e.g. E2A), class II bHLH E-box (e.g. TAL1) and GATA motifs, which are all components of the LMO2 complex. The *Lmo2^−/−^*-specific DHSs on the other hand were enriched in LEF/TCF motifs. We next ranked the ratio of tag counts in the DHS between WT and *Lmo2^−/−^* samples and plotted the position of the enriched motifs in relation to the DHS (Figure 4C). All motifs were localized in the centre of the DHSs thus validating the approach. The distribution of motifs between specific and shared DHS revealed that TAL1 E-box motifs and GATA motifs were enriched in the WT-specific DHSs, in particular as bipartite motifs of either TAL1-GATA or TAL1-TAL1, the latter originally described *in vitro* by the Rabbitts lab (2,10). SP1, ETS, and CREB motifs were enriched in the shared DHSs and T-box motifs were found throughout. These finding were confirmed by performing bootstrapping analysis on the top 10% of the DHSs ranked by WT/*Lmo2^−/−^* ratio, showing co-localisation of motifs for E2A, TAL1 and GATA in this DHS subset (Supplementary Figure S5).

### ChIPseq analysis shows a limited number of strong binding sites for the LMO2 complex

**Figure 5.**
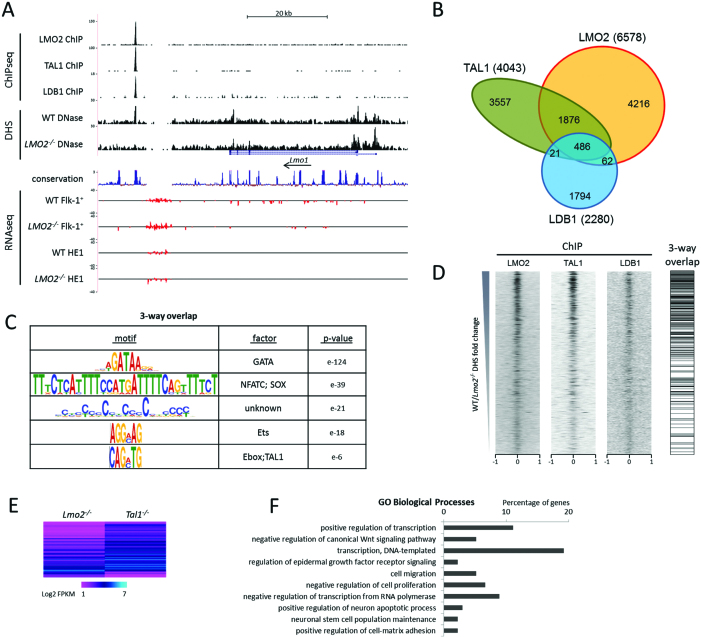
Binding profiles of LMO2, TAL1 and LDB1 in Flk-1^+^ cells. (**A**) Screenshot from the UCSC browser showing LMO2, TAL1 and LDB1 binding profiles, DNaseI accessibility in WT and *Lmo2^−/−^* Flk-1^+^ cells, and RNAseq of WT and *Lmo2^−/−^* Flk-1^+^ and HE1 cells at the *Lmo1* locus. Uniform y-axis scales were used for all RNAseq tracks and for the DNaseI WT to *Lmo2^−/−^* pairwise comparison. (**B**) Three-way Venn diagram showing the intersection of LMO2, TAL1 and LDB1 ChIPseq data obtained from Flk-1^+^ cells. (**C**) *De novo* motif analysis showing enriched motifs within the 486 peaks that are common for all three transcription factors. (**D**) Ranking of the ChIPseq data according to the WT/*Lmo2^−/−^* ratio as described in Figure 4 showing read density in a window from –1 kb to +1 kb around the centre of the DHSs, flanked by a density plot indicating the positions of three-way overlaps. (**E**) Log_2_ FPKM expression data of significantly differentially expressed genes between *Lmo2^−/–^* and *Tal1^−/−^* Flk-1^+^ cells, associated with TAL1 ChIPseq peaks. Genes were ranked according to their FPKM fold change. (**F**) Gene ontology enrichment analysis for biological process was performed on the 135 genes as identified in E. Terms were ordered according to their Modified Fisher Extract *P*-value, only terms with *P* < 0.05 were considered significant, and the top 10 are shown.

To examine, whether the loss of DHSs in *Lmo2^−/−^* HBs was due to the loss of the LMO2 complex, we determined the LMO2 targets in WT Flk-1^+^ cells by ChIPseq analysis (Figure 5A, Supplementary Figure S6A). Using antibodies recognising LMO2, TAL1 and LDB1 we detected 6578, 4043 and 2280 peaks, respectively. The intersection of the data sets showed that the three-way overlap was restricted to 486 peaks (Figure 5B). Motif analysis of these high confidence LMO2-complex binding sites revealed that the GATA motif was the most significantly overrepresented (Figure 5C). In addition we found several other associated motifs, such as ETS and E-box motifs. Ranking the ChIP results according to the DHSs fold change as shown in Figure 4, it was clear that the strongest ChIP signal occurs at the top for each of the ChIPseq samples and that the three-way overlap was highly enriched for WT specific DHSs (Figure 5D). One example of a target element where all three factors bind together, and where the DHS is severely affected in *Lmo2^−/−^* Flk-1^+^ cells, is the enhancer of the *Lmo1* gene (Figure 5A) (51). Taken together, our analyses show that (i) the loss of LMO2 leads to a loss of specific cis-regulatory elements, and (ii) that those elements are characterized by the presence of co-localizing motifs that bind known members of the LMO2 complex.

In order to find genes that TAL1 regulates in the absence of LMO2, we combined the *Lmo2^−/−^* and *Tal1^−/−^* gene expression data shown in Figure 3 with the TAL1 ChIPseq data. We identified the nearest genes of the TAL1 peaks with significantly different gene expression between the *Lmo2^−/−^* and *Tal1^−/−^* Flk-1^+^ cells, which resulted in a list of 135 genes. The majority of these genes (85%) had higher expression in the *Tal1^−/−^* cells implying that TAL1 acts as a suppressor in the absence of LMO2 (Figure 5E). Gene ontology analyses of biological processes revealed that among TAL1 targets many are transcription factors and suppressors of the canonical Wnt pathway (Figure 5F).

### TAL1 and LDB1 binding is redistributed in the absence of LMO2

**Figure 6.**
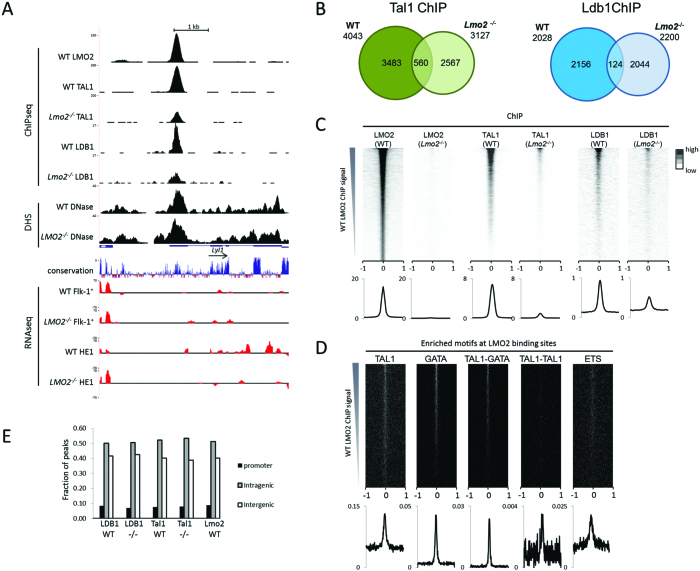
LMO2, TAL1 and LDB1 binding profiles in WT and *Lmo2^−/−^* haemangioblasts. (**A**) Screenshot from the UCSC browser showing LMO2, TAL1 and LDB1 binding profiles and DNaseI accessibility in WT and *Lmo2^−/−^* Flk-1^+^ cells, and RNAseq of WT and *Lmo2^−/−^* Flk-1^+^ and HE1 cells at the *Lyl1* locus. Uniform y-axis scales were used for all RNAseq tracks and for each WT to *Lmo2^−/−^* pairwise comparison. (**B**) Venn diagrams showing the overlap of the TAL1 and LDB1 ChIPseq peaks obtained from WT or *Lmo2^−/−^* Flk-1^+^ cells. (**C**) ChIPseq data for LMO2, TAL1 and LDB1 in WT and *Lmo2^−/−^* Flk-1^+^ cells, ranked according to the WT LMO2 ChIP signal, showing read density in a window from –1 kb to +1 kb around the centre of the LMO2 peaks. Below each matrix an average profile shows the distribution of the ChIP signal throughout the matrix. (**D**) Motif distribution matrices ranked according to the WT LMO2 ChIP signal, with average distribution profiles below. (**E**) Fractionation of peaks of each of the ChIPseq datasets on basis of their position in relation to genes.

We next investigated the localisation of LMO2, TAL1 and LDB1 in the absence of LMO2 by performing ChIPseq in *Lmo2^−/−^* Flk-1^+^ cells and compared these data to the WT-derived data sets (Figure 6A). The LMO2 ChIP in *Lmo2^−/−^* Flk-1^+^ cells resulted in a track without peaks, whereas 3127 peaks were called in *Lmo2^−/−^* samples using the TAL1 antibody and 2200 peaks for the LDB1 antibody (Figure 6B). We first ranked the ChIPseq peaks according to the WT LMO2 ChIP signal (Figure 6C) and compared the signals to the corresponding TAL1 and LDB1 peaks. This comparison demonstrated that the strongest WT LMO2 peaks were mirrored by the strongest TAL1 and LDB1 peaks. When looking at less strong LMO2 peaks, there was less overlap. However, these sites do show accumulation of reads in the TAL1 and LDB1 ChIP (lower part of Figure 6C), albeit not sufficiently to be recognised as peaks by data analysis. The LMO2 binding sites were enriched for the same motifs as shown for the WT-specific DHSs, i.e. TAL1, GATA, TAL1-GATA, TAL1-TAL1, as well as for ETS motifs (Figure 6D), whereas transcription factor binding motifs that were found enriched at non WT-specific DHSs were not enriched at the LMO2 peaks (Supplementary Figure S6B). Comparison of the WT ChIP data with those from the *Lmo2^−/−^* Flk-1^+^ cells showed that TAL1 and LDB1 binding was largely lost, with significant levels only being left at those binding sites where LMO2 occupancy was the strongest in WT (Figure 6C). GATA2 ChIP experiments indicated that also GATA2 binding was reduced in the absence of LMO2, however, the DNA pulldown we achieved was insufficient for genome wide analysis (Supplementary Figure S6A). The LMO2 peak present on the first exon of the *Lyl1* gene was one of the strongest in WT cells and serves as a good example of a position where TAL1 and LDB1 binding is clearly observed in the absence of LMO2 (Figure 6A). Peaks at the *Gata1* and *Nfe2* genes are representative of the majority of binding sites, which show very little or absent residual binding without LMO2 (Supplementary Figure S6C). Approximately half of the peaks within each of the ChIPseq data sets fall within intragenic and promoter regions (Figure 6E) in line with the chromatin nucleating function of LDB1, bringing distant regulatory elements in the vicinity of the gene they are regulating (3,4).

We observed that TAL1 and LDB1 binding in the *Lmo2^−/−^* HBs was extensively redistributed to new sites (Figure 6B). As LDB1 has multiple other potential binding partners in these cells, such as LMO4, LHX, OTX proteins (6,10,52), its changed distribution in the absence of LMO2 could be anticipated. In contrast, the redistribution of TAL1 binding in the *Lmo2^−/−^* was more surprising as it has been shown that TAL1 is capable of binding DNA in the absence of LMO2 (2,53). In order to further analyse this, we ranked the TAL1 ChIPs of WT and *Lmo2^−/−^* cells based on their signal ratio (Supplementary Figure S7A), which indeed showed a clear population of TAL1 peaks exclusive for *Lmo2^−/−^* cells. The average TAL1 ChIP signal distribution showed that TAL1 peaks in the *Lmo2^−/−^* cells have a lower intensity than in WT cells (Supplementary Figure S7B). Additionally, TAL1 peaks specific to the *Lmo2^−/−^* cells did not overlap with the WT LMO2 ChIP signal, or increased accessibility of the chromatin. Analysis of the E-box and GATA motif distribution indicated a striking reduction of E-box motifs and absence of GATA motifs at these sites (Supplementary Figure S7A). Our results suggest that the TAL1/LMO2 interaction is essential for the correct TAL1 DNA binding and thereby for the positioning of TAL1/LMO2/LDB1 complex. Experiments where LMO2 deficiency was corrected by an inducible LMO2 transgene showed a clear rescue of both LMO2 and TAL1 binding at target sites (Supplementary Figure S8).

### Strong LMO2 binding sites correspond to differentially expressed genes

**Figure 7.**
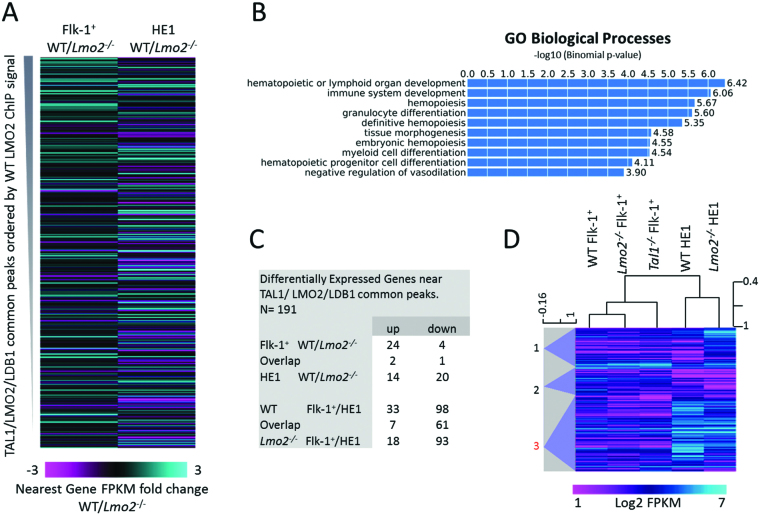
High-affinity LMO2 binding sites in the WT hemangioblasts are in the vicinity of genes that are upregulated in WT HE. (**A**) Heatmap of the FPKM fold change between the WT and *Lmo2^−/−^* for the genes nearest to the LMO2 ChIPseq peaks, at the Flk-1^+^ (left) and HE1 (right) stages. FPKM ratios are ranked according to the WT LMO2 ChIPseq signal. Scale bar represents colour index for the FPKM fold change. (**B**) GREAT gene ontology enrichment analysis for biological process performed on genomic regions of the triple peaks. (**C**) Table showing the number of genes associated with LMO2 ChIPseq peaks that have increased or decreased expression (>4-fold) in WT compared to *Lmo2^−/−^* cells. (**D**) Heat map of hierarchically clustered genes that associate with common TAL1, LMO2 and LDB1 ChIPseq peaks. These genes are either the nearest 5′ or 3′ gene, or contain the peak within the gene body. Scale bar represents colour index for the log_2_ FPKM values. Self-organizing tree analysis identified three clusters, which are numbered 1 to 3.

We next correlated ChIPseq binding data with differential gene expression. To this end, we determined the WT/*Lmo2^−/−^* ratio of gene expression of the nearest genes to the LMO2/TAL1/LDB1 triple peaks, in HBs and HE1 and ranked them according to the WT LMO2 peak signal intensity (Figure 7A). There was a clear positive correlation between the top triple peaks and the gene expression of the nearest genes in HBs, indicating that these genes were particularly sensitive to the absence of the LMO2 complex. Less strong peaks correlated less often with changes in gene expression. In comparison, the correlation between the HB triple peaks and the gene expression at the HE1 stage showed a much more extensive effect on gene expression, which was along all the peaks and correlated with both up and down regulation (Supplementary Figure S9). Gene ontology analysis of biological processes for these genomic coordinates revealed almost exclusively haematopoietic processes (Figure 7B). The total number of genes that were determined to be significantly upregulated or downregulated is shown in Figure 7C. Hierarchical clustering of these gene expression levels, including the gene expression from *Tal1^−/−^* HBs, resulted in three clusters (Figure 7D). Cluster 3 contained genes that failed to upregulate in *Lmo2^−/−^* HE1. This cluster contained a large number of genes encoding transcription factors with known involvement in haematopoietic development, such as *Rxra, Stat3, Stat5b, Ebf1, Nfatc1, Gata2, Sox7, Runx1t1* (ETO) and *Pbx1*. The lack of LMO2 and absence of haematopoietic differentiation therefore correlates with the absence of a haematopoiesis-specific transcriptional program.

## DISCUSSION

*Lmo2* is one of few transcriptional regulators which are part of the very core of blood stem/progenitor cell identity. LMO2 is essential for both primitive erythropoiesis and definitive haematopoiesis (25,26). Moreover, LMO2 is required for reprogramming of committed blood cells to induced haematopoietic stem cells (54). Failure to repress *Lmo2/Tal1* or reinstatement of their expression in lineage-committed cells induces stem cell properties (55,56). Recently, we showed that LMO2, in combination with TAL1, was at the start of the generation of a dynamic gene regulatory network programming haematopoietic specification, using *in vitro* ES cell differentiation (37). With the same differentiation system, we now used *Lmo2* and *Tal1* knockout cell lines to gain further understanding of the precise role of LMO2 at these initial stages of haematopoietic development. We found that *Lmo2* expression was established during the development of HBs and that the generation of *Lmo2^−/−^* Flk-1^+^ cells was not affected. However, the progression from HB to HE occurred with reduced efficiency and *Lmo2^−/−^* HE could only give rise to a small number of primitive haematopoietic progenitors. Therefore, *Lmo2^−/−^* cells were not blocked at one developmental stage of haematopoietic specification, but had a progressive defect. These observations are in agreement with the phenotype observed in mice.

Although WT and *Lmo2*^−/−^ ES cells have a similar capacity to produce Flk-1^+^ cells, our experiments showed that LMO2 exerts its function already at the HB stage by positioning the LMO2 complex to regulatory elements important for the establishment of the haematopoietic program. As the peaks identified by the three-way overlap of the LMO2, TAL1 and LDB1 ChIPseq data were the sites with the highest number of reads in each of the independent samples, we conclude that these regulatory elements exhibit the highest affinity for this complex. In the absence of LMO2, binding of TAL1 only remained at the strongest of these binding sites, albeit at a lower level, whereas the majority of TAL1 binding was found at novel binding sites. It has previously been shown that the interaction of LMO2 with TAL1 directly increases the stability of TAL1/E2A heterodimer (57). Experiments with TAL1 DNA-binding deficient mutants showed that DNA-binding activity of TAL1 was dispensable for specification of haematopoietic development (53), only when the interaction of the TAL1 HLH domain with LMO2 was retained (57). In addition, in the presence of LMO2, TAL1 could associate with chromatin in the absence of its DNA binding domain, albeit with a lower affinity (58). Our motif analysis of the three-way overlap of LMO2, TAL1 and LDB1 ChIP indicated a very strong enrichment of GATA binding motifs (Figure 5). Taken together, this indicates that the interaction through LMO2 plays an important role in directing TAL1 to the correct binding sites, whereas without LMO2, TAL1 has lower DNA binding activity and specificity. Comparison of our data to previously published LDB-1 ChIPseq data in Flk-1^+^ cells (28) and in Lin^−^ bone marrow (59) showed that the overlap is particularly enriched for those LDB1 peaks that are part of the three-way overlap (83% and 51% of LMO2, TAL1 and LDB1 ChIP peaks respectively). In addition, our and previous studies identify the same list of target genes (e.g. Tal1, Gata2, Runx1, Lmo2, Runx2t2 and Sox7). Intersecting the lists of differentially expressed genes reported by Mylona *et al.* indicated that the *Ldb1^−/−^* was more similar to the *Tal1^−/−^*, in line with the developmental block occurring earlier than in the *Lmo2^−/−^*.

Meta-analysis of the LMO2 complex binding sites identified an overrepresentation of regulatory elements important for haematopoietic development. These included transcription factor genes known to be important at the HB stage (e.g. *Fli1* (52,59), *Gata2* (32), *Erg* (55)), genes that are important for the HE and endothelial-haematopoietic transition (e.g. *Gfi1* (60)), those with known haematopoietic stem cell function (e.g. *Lyl1* (61), *Stat3* (62)), and transcription factors with more lineage restricted functions (e.g. *Ebf1* (63), *Gata1* (64), *Nfe2* (65)). Our findings indicate that already at the HB stage, LMO2 primes transcription factor genes which will be upregulated at later stages of haematopoiesis and thus regulates all steps of haematopoietic development.

TAL1 and LMO2 are clearly intimately linked in their function and their knock-out phenotypes are similar. *In vitro* differentiation of *Lmo2^−/−^* cells demonstrated less severe differentiation defects than observed in *Tal1^−/−^*(20). *Lmo2^−/−^* Flk-1^+^ cells showed reduced *Tal1* mRNA and protein levels, whereas in *Tal1^−/−^* Flk-1^+^ cells *Lmo2* mRNA was completely lost, indicating that TAL1 is required for the initiation of *Lmo2* expression. This is in contrast to studies in zebrafish, which showed that the absence of TAL1 or LMO2 did not influence the mRNA expression of the other (66,67), although injection of *Tal1* mRNA in zebrafish embryos could induce *Lmo2* expression in somitic paraxial mesoderm (68).The residual DNA binding by remaining TAL1 protein is likely the reason why a fraction of *Lmo2*^−/−^ cells differentiate from HB to HE1 and give rise to primitive haematopoietic progenitors. The comparison of *Lmo2^−/−^* to WT HE may serve to define the differences between primitive and definitive HE of the mouse. Also, it was shown that *Tal1^−/−^* cells do not upregulate *Runx1* expression and that the differentiation defect could be partially rescued by introduction of RUNX1 (69). In contrast, *Lmo2^−/−^* HE1 does express *Runx1*, indicating that its upregulation is not adversely affected by the absence of LMO2. As RUNX1 is vitally important for the HE identity and the transition from HE to haematopoietic progenitors (38), this could represent a crucial difference between *Lmo2^−/−^* and *Tal1^−/−^* cells.

The *in vitro* differentiation of ES cells to the haematopoietic fate with particular transcription factors knocked out, has served as a great tool to further our understanding of how transcriptional networks operate throughout differentiation programs (20,28,36,38,60,69,70). Several of these studies have used induced expression of transcription factors to understand their capacity to rescue the observed knock-out phenotype. Given that the *Lmo2^−/−^* phenotype is progressive through differentiation and that this seems reflected in the developmental spread of the affected target genes, multiple target genes may need to be induced before haematopoietic development is efficiently restored.

## ACCESSION NUMBER

Genome wide data reported in this study are available from the NCBI Gene Expression Omnibus portal (GEO: GSE99938).

## Supplementary Material

Supplementary Data
