# Supplementary material for: LMO2 is required for TAL1 DNA binding activity and initiation of definitive haematopoiesis at the haemangioblast stage
Source: Nucleic Acids Res. 2017 Jun 30;45(17):9874–88. doi: 10.1093/nar/gkx573 (PMC5622341; doi:10.1093/nar/gkx573)
Supplement: Supplementary Data [file gkx573_supp.zip › Supplementary material.pdf]

Supplementary Material and Methods

Generation of *Lmo2*$^{-/-}$ ES cell line with doxycycline-inducible LMO2 (iL2KO)

For the creation of the LMO2 rescue, we used the E14Tg2a-derived A2lox (kindly provided by Professor Kyba, Department of Pediatrics, University of Minnesota, USA) ES cell line. A2lox ES cells express the reverse tetracycline transactivator from the *Rosa26* locus and carry a cassette containing a tetracycline response element, loxP-lox2272 sites and the neomycinΔATG-resistance gene upstream of the *Hprt* gene. For targeting the *Lmo2* gene, we made use of the *Lmo2* targeting construct, harbouring a hygromycin resistance gene, as described in Yamada et al. (2000). We replaced the hygromycin resistance gene for the blasticidin resistance gene, creating a second targeting vector. Cells were first transfected with the blasticidin targeting construct and subsequently underwent selection using 8 µg/ml blasticidin (Thermo Fisher) and 2 µM ganciclovir (Sigma). Single colonies were picked and after confirmation of the *Lmo2*$^{+/-}$ genotype underwent a second round of targeting using the hygromycin targeting construct. Cells were selected using 300 µg/ml hygromycin B (Thermo Scientific) and 2 µM ganciclovir, after which single colonies were picked and the *Lmo2*$^{-/-}$ genotype confirmed. To introduce the *Lmo2* cDNA behind de doxycycline-inducible promoter, the mouse *Lmo2* cDNA, with an N-terminal HA-tag, followed by an IRES-GFP was subcloned into the p2Lox targeting vector. Cre / Lox recombination was used to insert p2Lox into the inducible locus as described by Kyba et al. (2003), followed by selection with 400 µg/ml G418 (Thermo Fisher) and picking of single colonies. For ChIP analysis, the resulting iL2KO cells were differentiated as described and, where indicated, cells were treated with 1 µg/ml doxycycline for 16 hours prior to harvesting of Flk-1^{+} cells.

Western blotting

Western blotting was performed as escribed, using antibodies raised against GATA2 (R&D systems AF2046), HEB (Santa Cruz sc-357), E2A (Santa Cruz sc-763) and RUNX1 (Abcam Ab23980).

Principal Component Analysis

Cuffnorm (2.2.1.1) was used on bam files for the FPKM normalisation. The Principal Component Analysis (PCA) was carried out on the FPKM values of each experiment and was calculated using “prcomp” function implemented in R (R Core Team, 2014) and the scatterplot3d R package was used for the PCA 3D plot.

References:

Yamada Y, Pannell R, Forster A, Rabbitts TH (2000) The oncogenic LIM-only transcription factor Lmo2 regulates angiogenesis but not vasculogenesis in mice. *PNAS* **97**, 320-324

Kyba M, Perlingeiro RC, Hoover RR, Lu CW, Pierce J, Daley GQ (2003) Enhanced hematopoietic differentiation of embryonic stem cells conditionally expressing Stat5. *PNAS* **100** Suppl 1, 11904–11910

R Core Team (2014). R: A language and environment for statistical computing. R Foundation for Statistical Computing, Vienna, Austria. URL http://www.R-project.org/.

Stanulovic et al. Figure S1

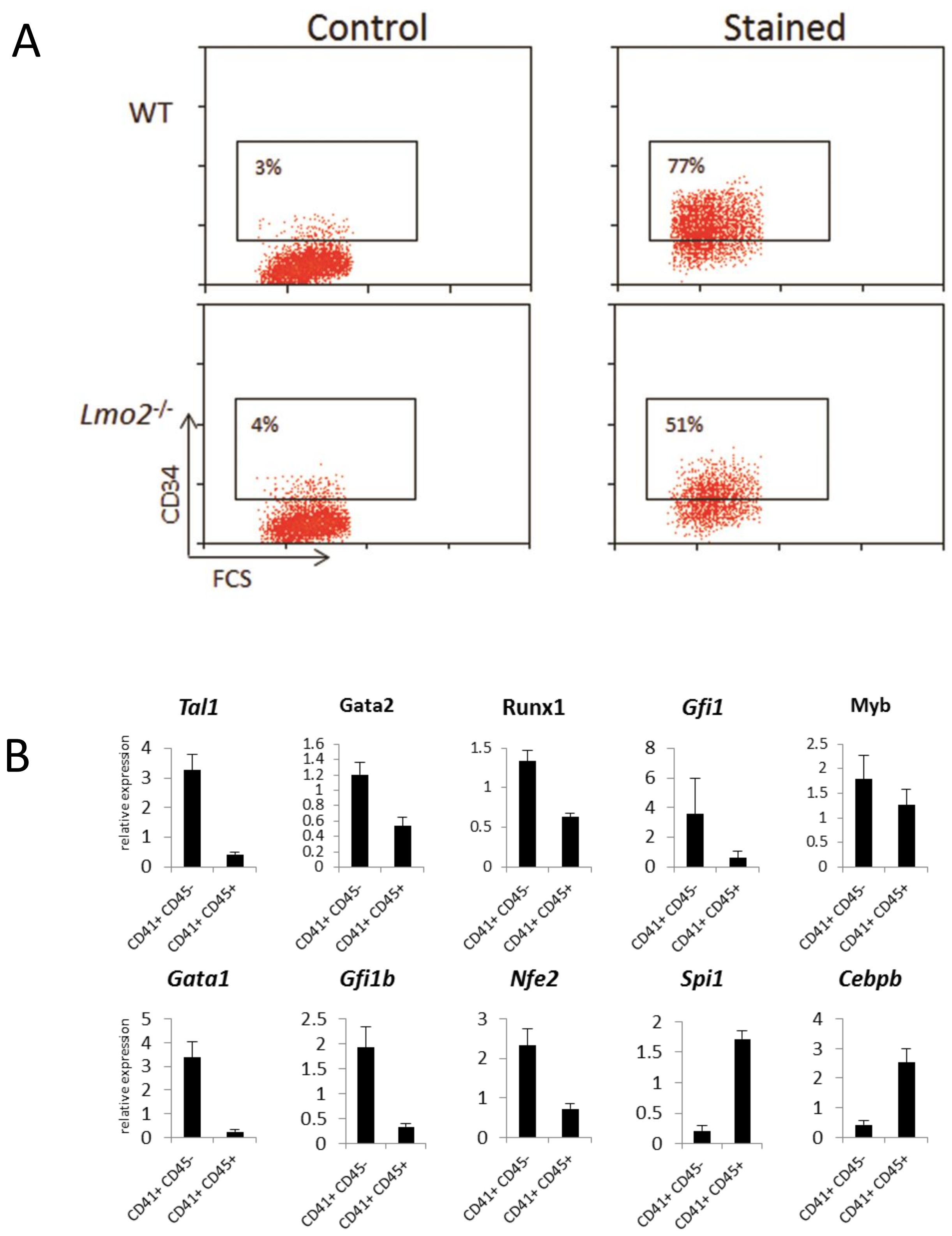

Stanulovic et al. Figure S2

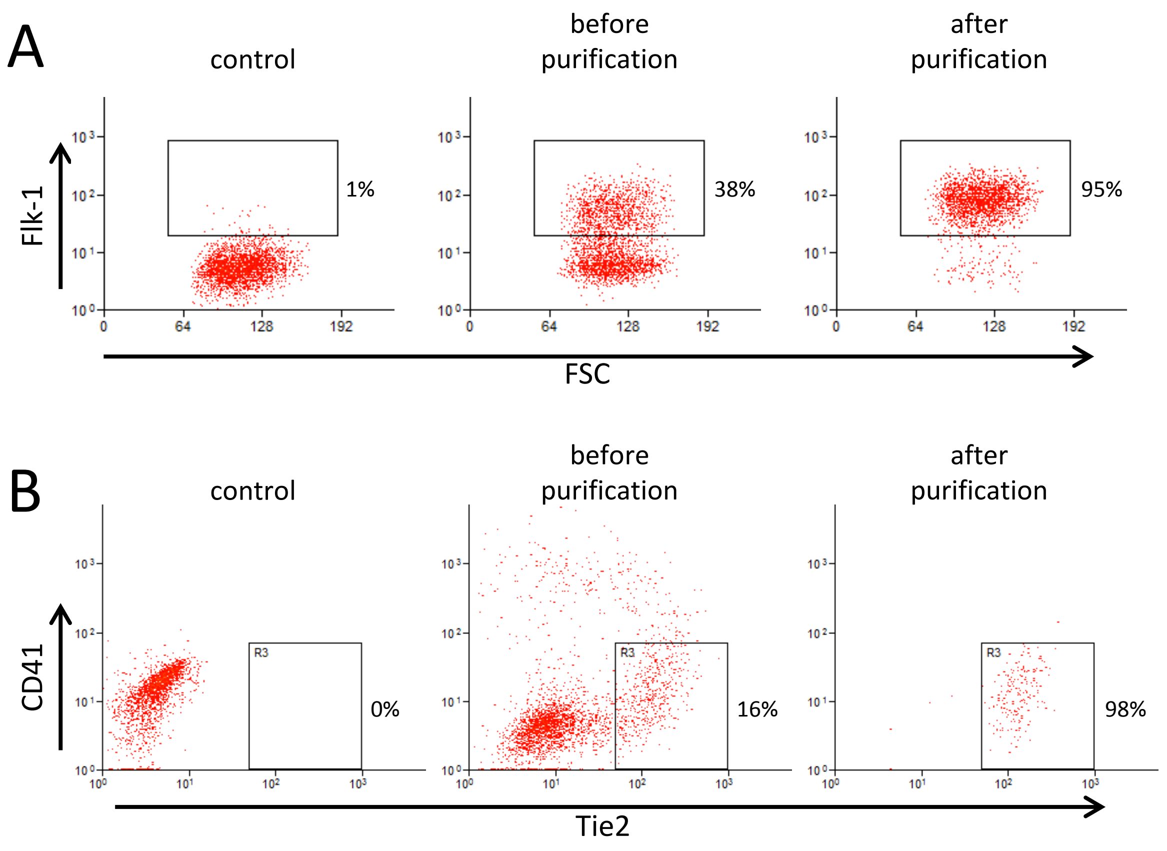

Stanulovic et al. Figure S3

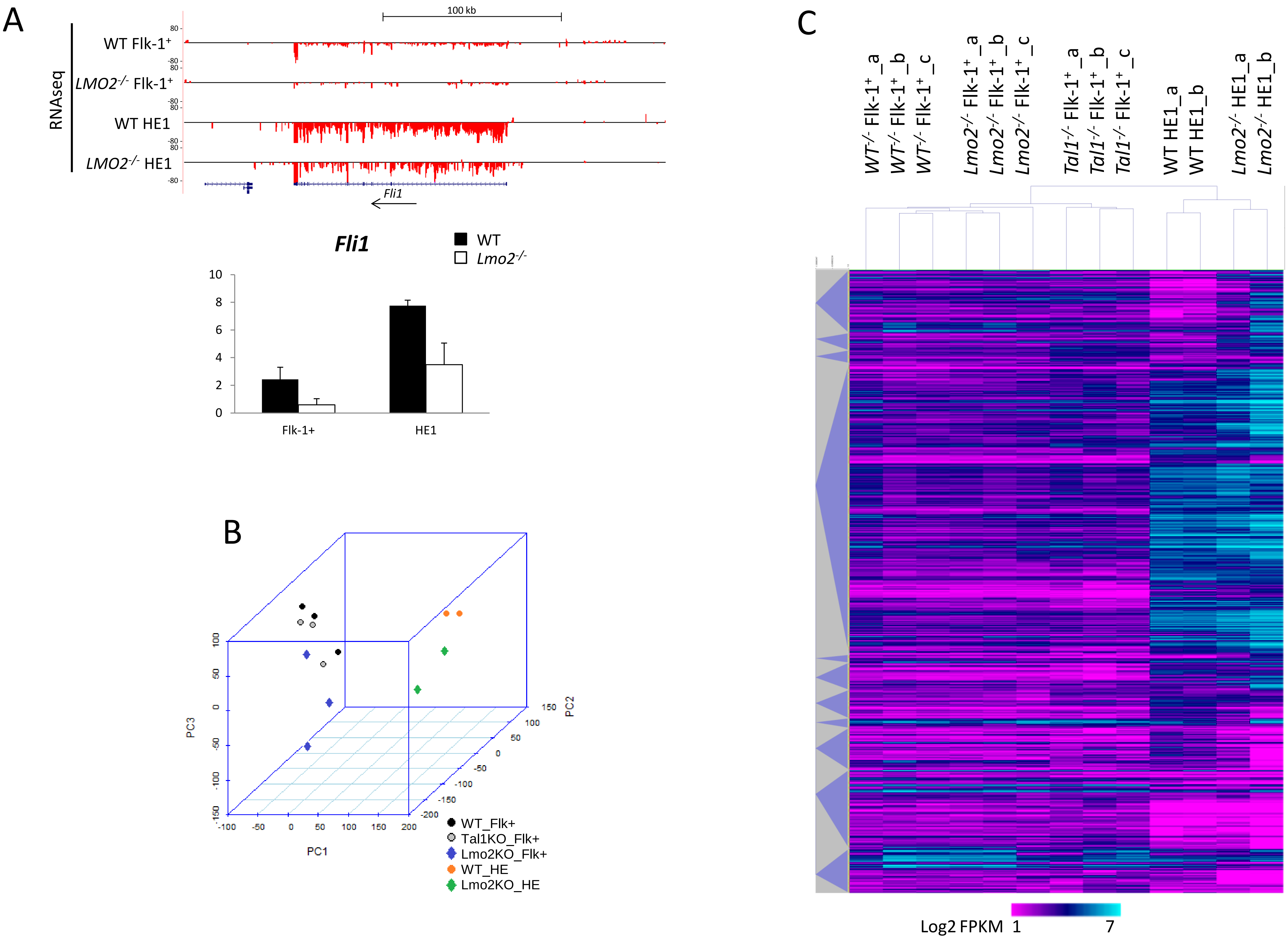

Stanulovic et al. Figure S4

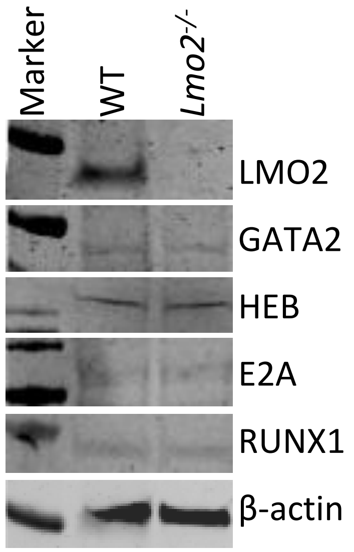

Stanulovic et al. Figure S5

Bootstrapping analysis; top 10% WT/*Lmo2*$^{-/-}$ DHSs

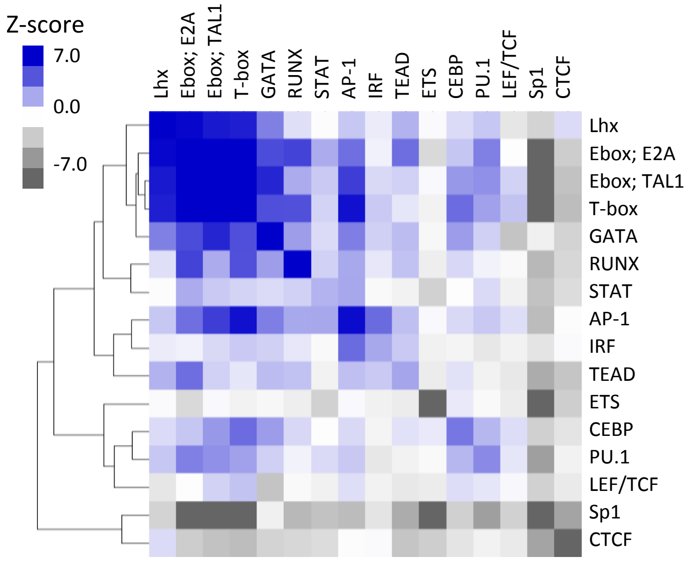

Stanulovic et al. Figure S6

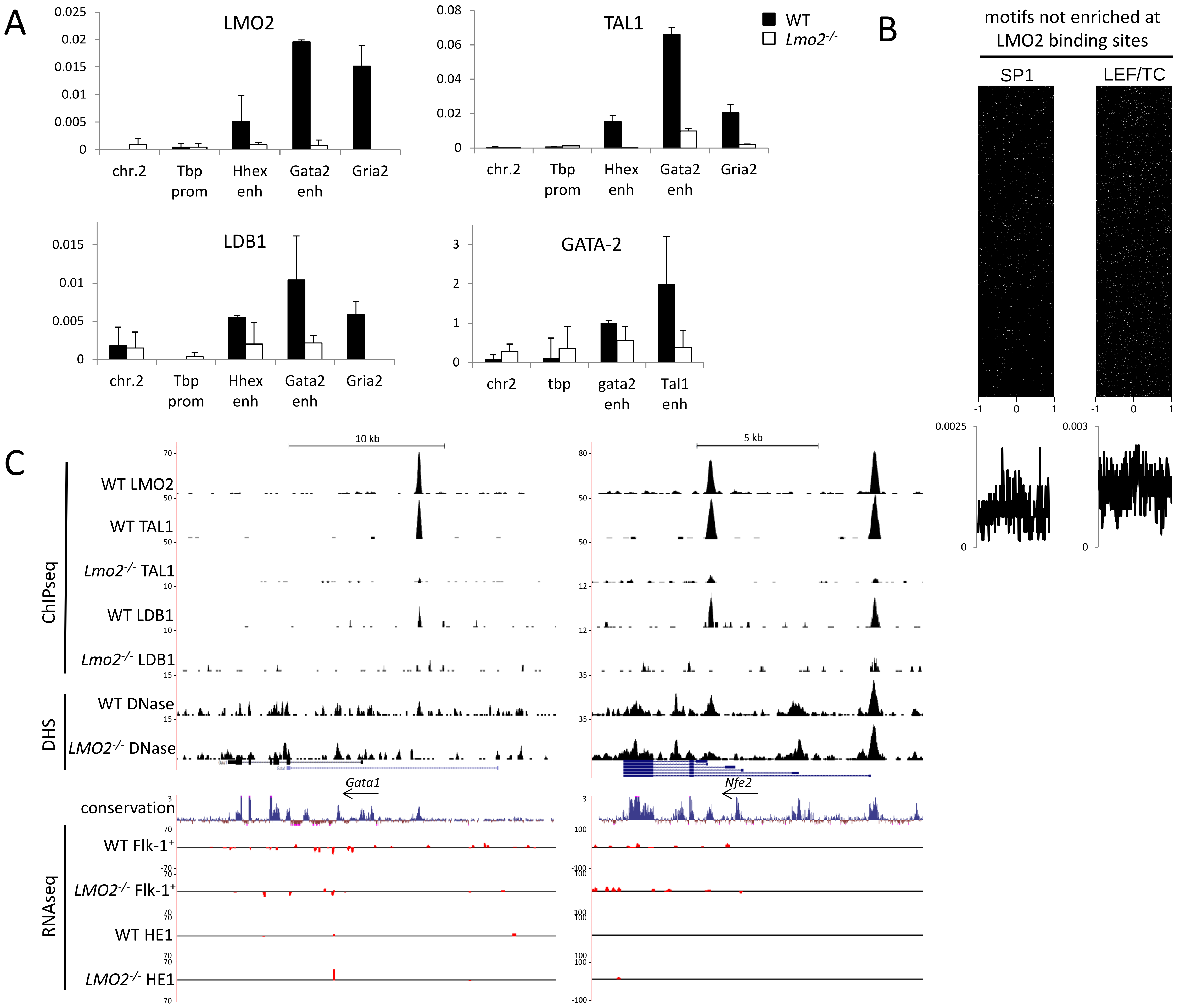

Stanulovic et al. Figure S7

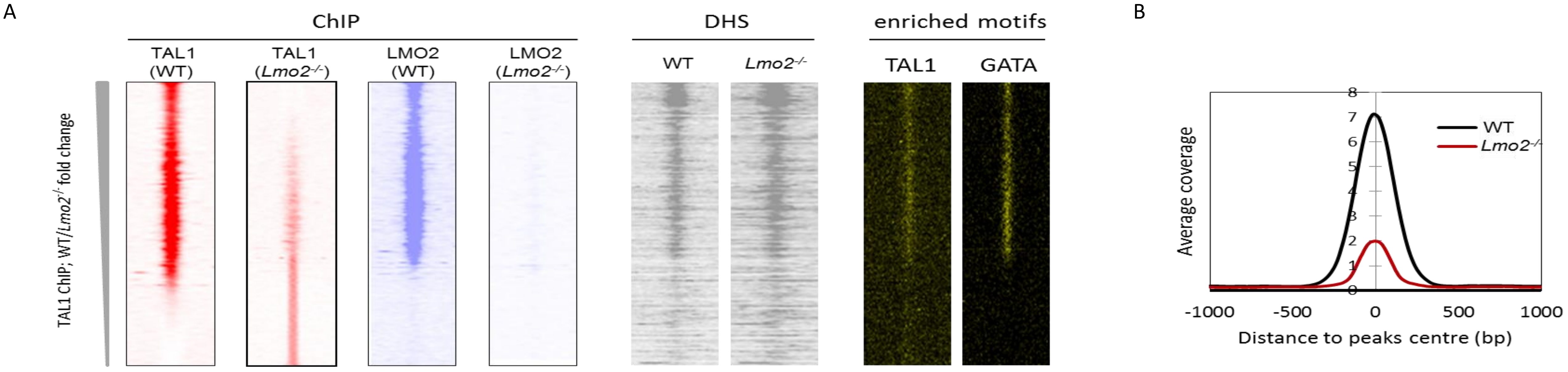

Stanulovic et al. Figure S8

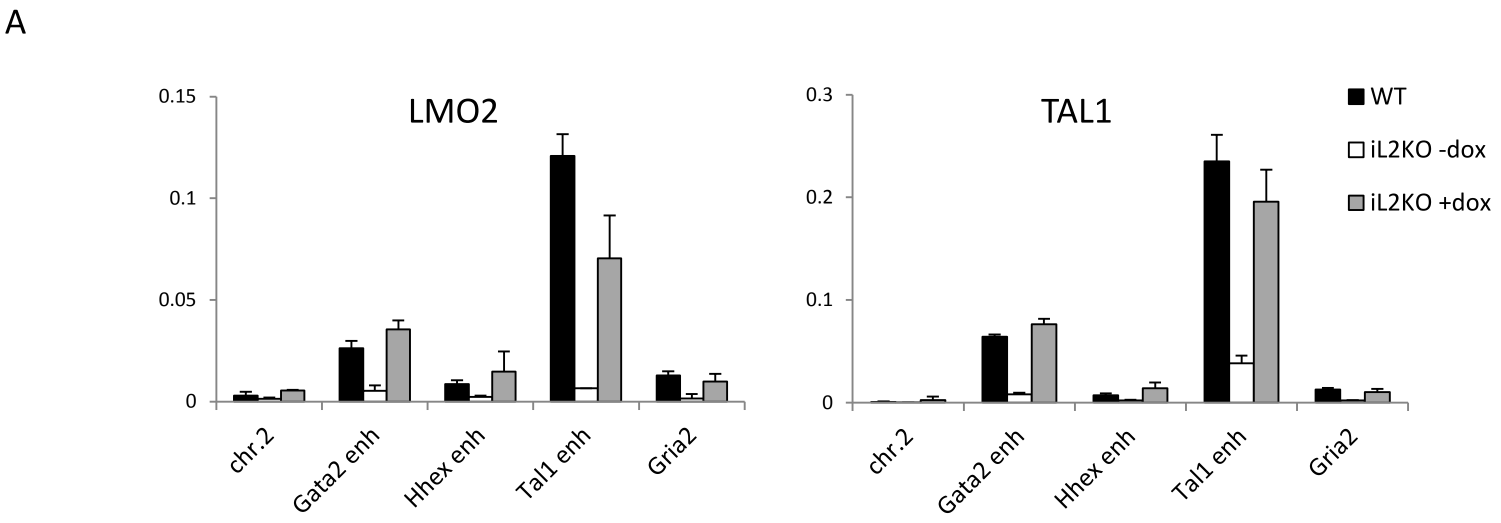

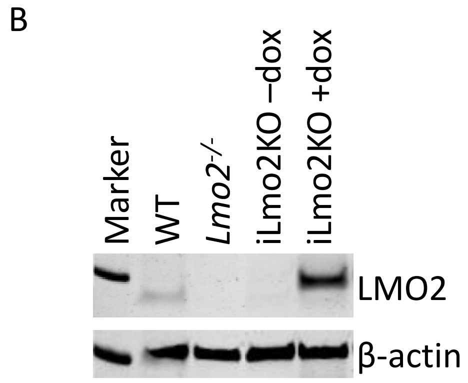

Stanulovic et al. Figure S9

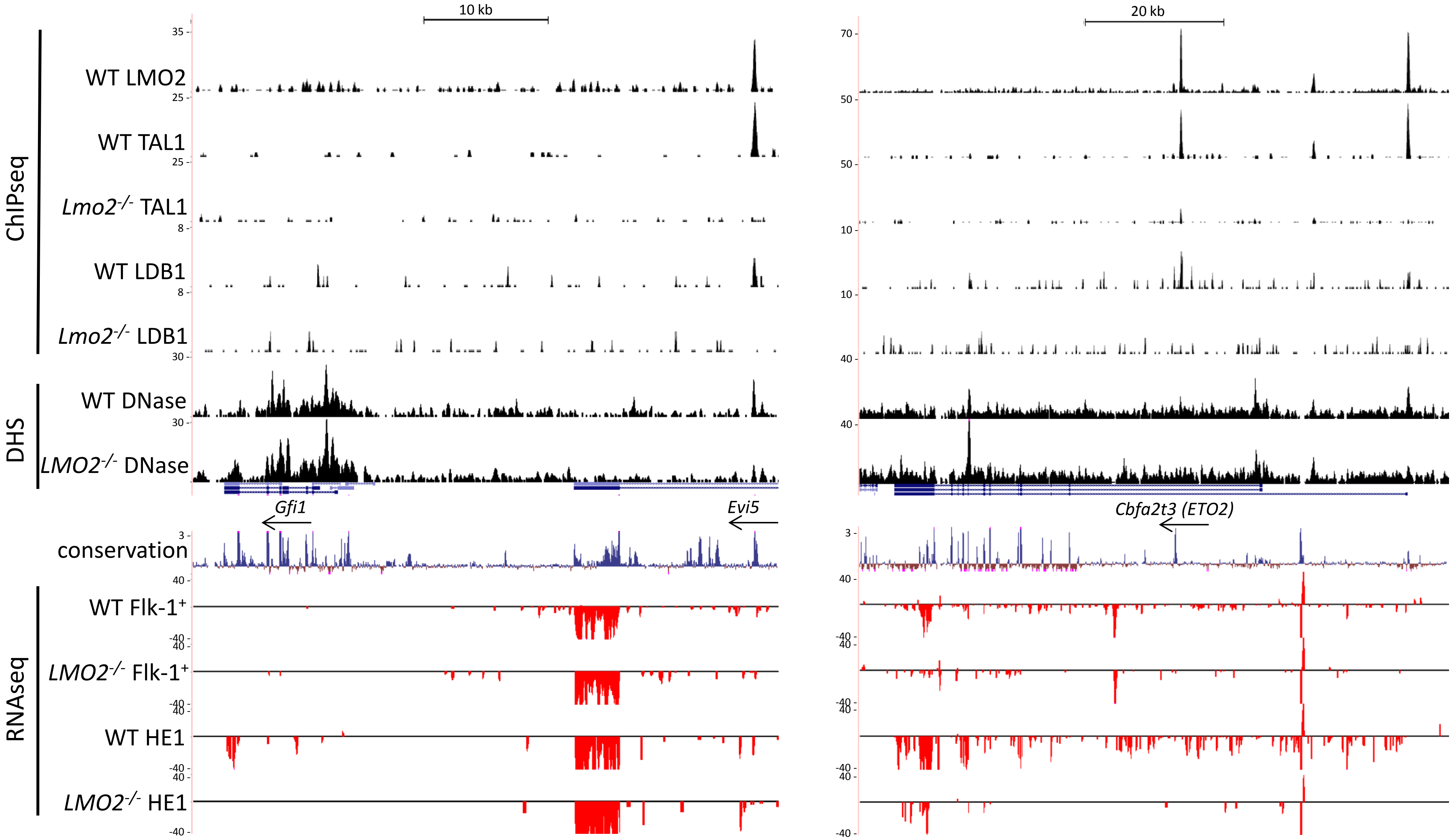

Figure S1. Gene expression analysis on heamatopoietic progenitors.

Relative expression levels of transcription factor genes in sorted populations of WT CD41^{+}; CD45^{-} (n=7) and WT CD41^{+}; CD45^{+} (n=6) haematopoietic progenitors were measured by qPCR in duplicate relative to *Hprt* ± SEM.

Figure S2. Purity confirmation of isolated populations

(A) EB-derived cells were stained for Flk-1 surface expression prior to and after MACS sorting. (B) Cells from day 2 blast culture were stained for Kit, Tie2, and CD41 surface expression and sorted. The profile before and after the FACS sort are shown.

Figure S3. Genome browser tracks and validation.

(A) UCSC browser tracks illustrating the distribution of reads over the *Fli1* gene. Uniform values for the y-axis were used for each genomic region and positive and negative values correspond to the direction of the transcripts. Gene expression levels of *Fli1* as measured by qPCR are shown below the plot. Data represent the mean of three independent samples measured in duplicate relative to *Hprt* ± SEM. **(B)** Principle component analysis of the thirteen RNAseq samples used in this study, based on the total normalized FPKM values. **(C)** Hierarchical clustering analysis of the thirteen RNAseq samples used in this study, based on the normalized FPKM values of differentially expressed genes.

Figure S4. Western blot analysis.

Protein levels of LMO2, GATA2, HEB, E2A, RUNX1 and β-actin as detected by western blotting using nuclear extract from WT and Lmo2$^{-/-}$ Flk-1^{+} cells.

Figure S5. Co-localisation of transcription factor binding sites.

Bootstrapping analysis showing the co-localisation of transcription factor binding motifs, using the top 10% of the DHS ranked by WT/*Lmo2*$^{-/-}$ ratio.

Figure S6. ChIP data analysis.

(A) ChIP qPCR experiments measuring the enrichment of LMO2, TAL1, LDB1 and GATA2 (αGATA-2; Santa Cruz sc-9008) enrichment at a heterochromatin region of Chr2 (negative control), the euchromatic *Tbp* promotor (negative control), *Hhex* intronic enhancer, *Gata2* intronic enhancer, *Gria2* intronic element and Tal1 downstream enhancer. Bars represent mean of three independent experiments, measured in duplicate ± standard deviation. **(B)** SP1 and LEF/TCF motif distribution matrices according to the WT LMO2 ChIP signal, with average distribution profiles below. **(C)** Two screenshots from the UCSC browser showing LMO2, TAL1 and LDB1 binding profiles and DNaseI accessibility in WT and *Lmo2*$^{-/-}$ Flk-1+ cells, and RNAseq of WT and *Lmo2*$^{-/-}$ Flk-1^{+} and HE1 cells at the *Gata1* and *Nfe2* loci. Uniform y-axis scales were used for all RNAseq tracks and for each WT to *Lmo2*$^{-/-}$ pairwise comparison.

Figure S7. TAL1 localisation in *Lmo2*$^{-/-}$ cells.

(A) TAL1 and LMO2 ChIPseq data, DHS, and enriched TAl1 and GATA motifs in WT and *Lmo2*$^{-/-}$ Flk-1^{+} cells, ranked according to the ratio of the TAL1 ChIP signal between WT and *Lmo2*$^{-/-}$ cells, each showing a window from -1kb to +1kb around the centre of the TAL1 peaks. **(B)** Average coverage of the reads at TAL1 peaks in WT (black line) and *Lmo2*$^{-/-}$ (red line) Flk-1+ cells.

Figure S8. Rescue of Lmo2-/- cells by inducible expression of LMO2.

(A) LMO2 and TAL1 ChIP analysis of Flk-1^{+} cells isolated from WT and iL2KO EBs. HA-LMO2 transgene expression was induced in iL2KO cells by treatment 1 μg/ml doxycycline for 16 hours prior to harvesting or vehicle as a control. Measurements were performed by qPCR at a heterochromatin region of Chr2 (negative control), *Hhex* intronic enhancer, *Gata2* intronic enhancer, *Gria2* intronic element and Tal1 downstream enhancer. Bars represent mean of three independent experiments, measured in duplicate ± standard deviation. **(B)** Western blot showing LMO2 and β-actin expression in the indicated cell types.

Figure S9. Screenshots of the *Gfi1* and *Cbfa2t3* loci.

Two screenshot from the UCSC browser showing LMO2, TAL1 and LDB1 binding profiles and DNaseI accessibility in WT and *Lmo2*$^{-/-}$ Flk-1^{+} cells, and RNAseq of WT and *Lmo2*$^{-/-}$ Flk-1^{+} and HE1 cells at the *Gfi1* and *Cbfa2t3* (encoding ETO2) loci. Uniform y-axis scales were used for all RNAseq tracks and for each WT to *Lmo2*$^{-/-}$ pairwise comparison.

Supplementary Table 1. Primers used in the qPCR experiments.

**PRIMERS FOR GENE EXPRESSION**		
TARGET	FORWARD 5’	REVERSE 3’
Gata2	GCCTGTGGCCTCTACTACAAGCT	CCGATTCCGGGTCTGGAT
Eomes	AAAGTCTTCCGGGACAACTA	TAATATCGGGCTTGAGGCAA
Erg	AGGAGCTGTGCAAGATGACA	TCAGATGTGGAAGGGGAGTC
Lmo2	ACGGAAATTGTGCAGGAGAG	ACCCGCATCGTCATCTCATA
Tal1	CCAACAACAACCGGGTGAAG	GCCGCACTACTTTGGTGTGAG
Fli1	GCTGTTGTCGCACCTCAGTTAC	TCGTGAGGACTGGTCTGTATGG
Runx1	AGATTCAACGACCTCAGGTTTG	CGGATTTGTAAAGACGGTGATG
Gfi1	GTGAGCCTGGAGCAACACAA	CTCTTGAAGCTCTTGCCACAGA
Gfi1b	AGACCTTGGCATGGAACACAAT	GACTCAGAGAGTGGTGATTCCC
Myb	TGAACCCTGAACTCATCAAAGGT	GACCAACGCTTCGGACCATAT
Gata1	GTCAGAACCGGCCTCTCATC	GTGGTCGTTTGACAGTTAGTGCAT
Spi1	CCATAGCGATCACTACTGGGATTT	TGTGAAGTGGTTCTCAGGGAAGT
Cebpb	GTTTCGGGACTTGATGCAATC	CGCAGGAACATCTTTAAGGTGAT
Nfe2	TCCTCAGCAGAACAGGAACAG	GGCTCAAAAGATGTCTCACTTGG
Hprt	AGCGTCGTGATTAGCGATGA	TTCCAAATCCTCGGCATAATG
**PRIMERS FOR CHIP ANALYSES**		
TARGET	FORWARD	REVERSE
Chr.2	AGGGATGCCCATGCAGTCT	CCTGTCATCAGTCCATTCTCC
Tbp	TGCAGTCAAGAGCGCAACTG	CACCGCTACCGGACTCGAT
Gata2	GGAGTTTCCTATCCGGACATC	AGTCGAGGTGGCTCTGAAAAC
Hhex	CTGACCCTTTCCGTTCATACA	AATCAGCAGCGTGCACTACTC
Gria2	ACCACCTGCAAATCTTCCCC	CTACCATTTCCGGCCTCCAG
Tal1	TGACCTACAGCTCTCCTCCC	AGTCTGAGATTAGGCCCCGT
